# Supplementary material for: Circulating CD138 enhances disease progression by augmenting autoreactive antibody production in a mouse model of systemic lupus erythematosus
Source: J Biol Chem. 2021 Aug 6;297(3):101053. doi: 10.1016/j.jbc.2021.101053 (PMC8405997; doi:10.1016/j.jbc.2021.101053)
Supplement: Supplemental Figures S1–S5 [file mmc1.pdf]

Circulating CD138 enhances disease progression by augmenting autoreactive antibody production in a mouse model of systemic lupus erythematosus

Lunhua Liu and Mustafa Akkoyunlu*

Laboratory of Bacterial Polysaccharides, Division of Bacterial Parasitic and Allergenic Products, Center for Biologics, Evaluation and Research, the US Food and Drug Administration, Silver Spring, Maryland.

Supporting information-Figures

Figure S1

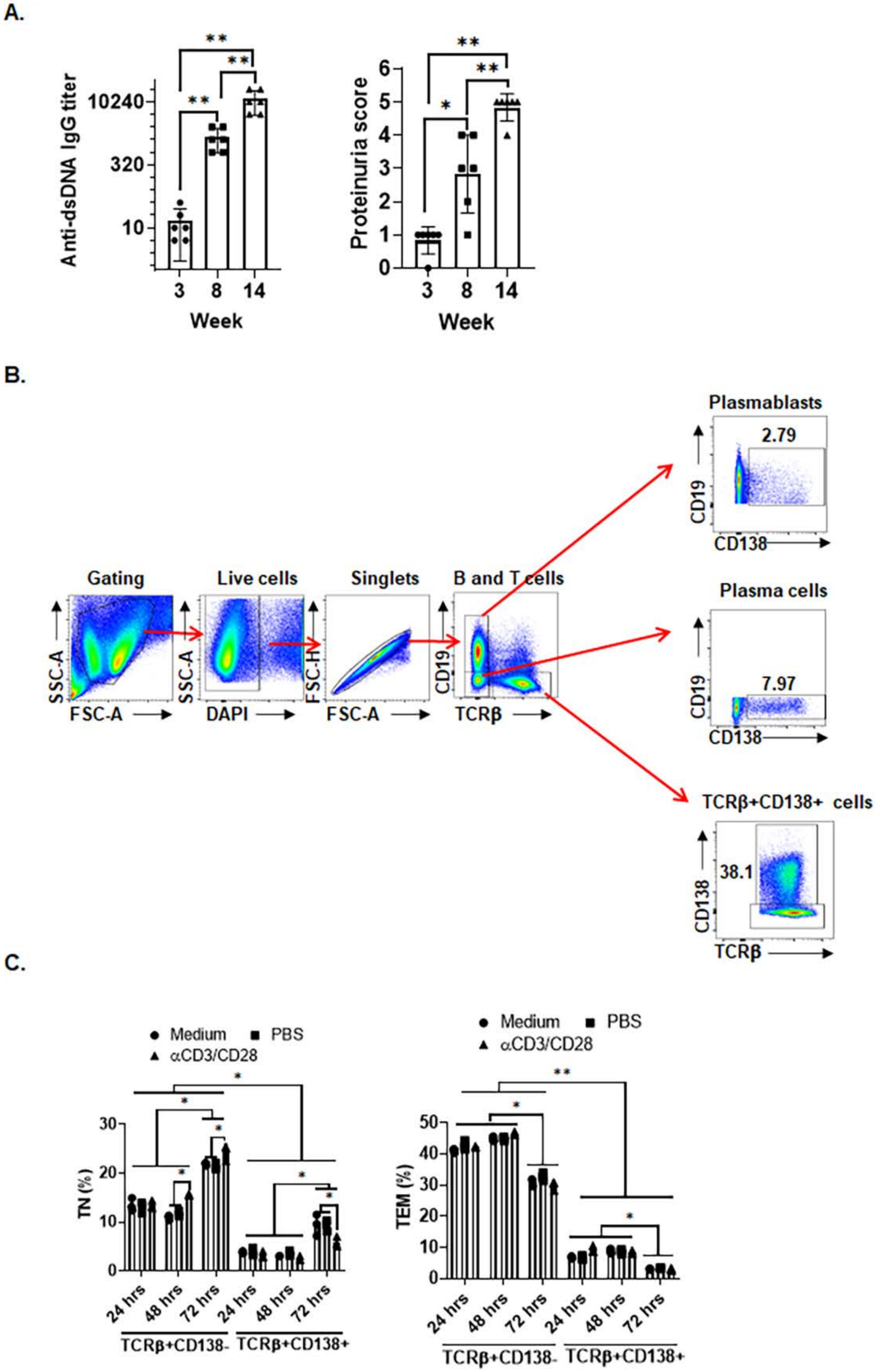

A.
Anti-dsDNA IgG titer
10240
320
10
3
8
14
Week
**
*
Proteinuria score
0
1
2
3
4
5
6
B.
Plasmablasts
2.79
CD19
CD138
Gating
Live cells
Singlets
B and T cells
SSC-A
FSC-A
DAPI
FSC-H
CD19
TCRβ
Plasma cells
7.97
TCRβ+CD138+ cells
38.1
CD138
C.
Medium
PBS
αCD3/CD28
TN (%)
TEM (%)
0
10
20
30
40
50
24 hrs
48 hrs
72 hrs
TCRβ+CD138-
TCRβ+CD138+

Figure S1. Lupus disease parameters, CD138-expressing cell subsets and memory T cell frequency after stimulation.
A. Serum anti-dsDNA IgG titers and proteinuria scores of MRL/Lpr mice from different ages are shown. Mean ± SD of 6 mice from two independent experiments are plotted. ***B.*** Singlets and live splenocytes were analyzed by FACS using B, T and plasma cell surface markers. ***C.*** Purified TCRβ+CD138- and TCRβ+CD138+ cells were treated with αCD3/CD28 antibodies for indicated durations, and TN and TEM cell percentages were measured by FACS. Mean ± SD of 3 mice from three independent experiments are plotted. *p<0.05, **p<0.01.

Figure S2

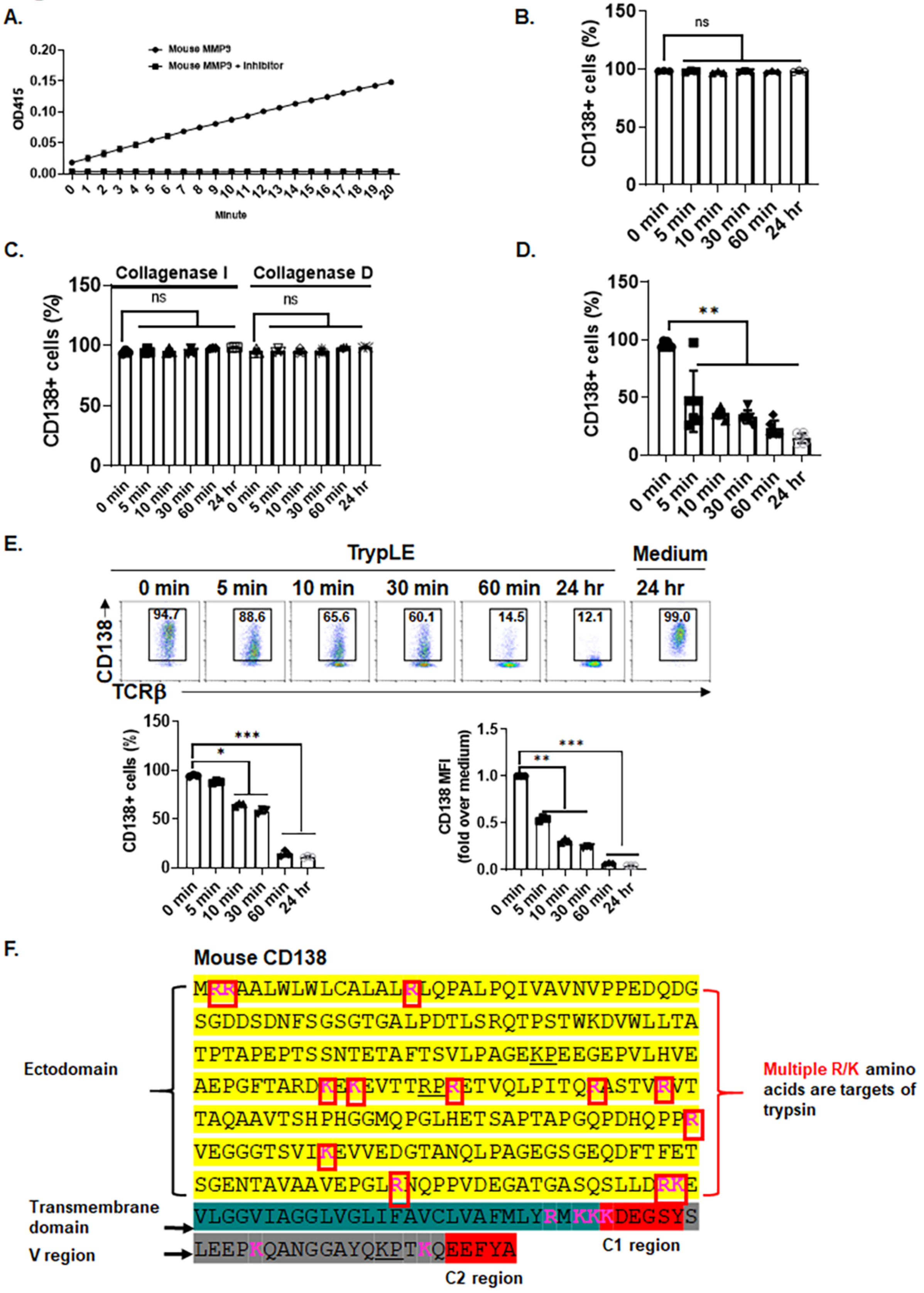
A.
Mouse MMP9
Mouse MMP9 + Inhibitor
OD415
Minute
B.
CD138+ cells (%)
ns
0 min
5 min
10 min
30 min
60 min
24 hr
C.
Collagenase I
Collagenase D
ns
CD138+ cells (%)
D.
CD138+ cells (%)
**
E.
TrypLE
Medium
0 min
5 min
10 min
30 min
60 min
24 hr
24 hr
94.7
88.6
65.6
60.1
14.5
12.1
99.0
CD138
TCRβ
CD138+ cells (%)
*
***
CD138 MFI
(fold over medium)
**
***
F.
Mouse CD138
MRRAALWLWLCALALRLQPALPQIVAVNVPPEDQDG
SGDDSDNFSGSGTGALPDTLSRQTPSTWKDVWLLTA
TPTAPEPTSSNTETAFTSVLPAGEKPEEGEPVLHVE
AEPGFTARDKEKEVTTRPRETVQLPITQRASTVRVT
TAQAAVTSHPHGGMQPGLHETSAPTAPGQPDHQPPR
VEGGGTSVIKEVVEDGTANQLPAGEGSGEQDFTFET
SGENTAVAAVEPGLRNQPPVDEGATGASQSLLDRKE
VLGGVIAGGLVGLIFAVCLVAFMLYRMKKKDEGSYS
LEEPKQANGGAYQKPTKQEEFYA
Ectodomain
Transmembrane domain
V region
C1 region
C2 region
Multiple R/K amino acids are targets of trypsin

Figure S2. CD138 on lupus T cells is resistant to MMP9 but sensitive to trypsin cleavage. ***A.*** The activity of mouse MMP9 was verified with the MMP9 colorimetric drug discovery kit using MMP9 specific inhibitor or not. Mean of two independent experiments are plotted. ***B - D.*** Purified TCRβ+CD138+ cells from 12 weeks old MRL/Lpr mice were treated with active MMP9 **(*B*)** collagenase I and D **(*C*)**, or trypsin **(*D*)** for indicated durations and the percentages of CD138-expressing cells were measured by FACS. Mean ± SD of three independent experiments are plotted. ns, not significant. ***E***. Purified TCRβ+CD138+ cells from 12 weeks old MRL/Lpr mice were treated 10% TrypLE for indicated time, and CD138 positive cells percentages and CD138 expression levels were measured by FACS. Mean ± SD of three independent experiments are plotted. ***F***. Extracellular, transmembrane, and intracellular domains of murine CD138 were labeled with yellow, turquoise, and green colors, respectively. The trypsin targets, lysine (R) and arginine (K) amino acids in the extracellular domain are highlighted in red box. Lysine and arginine residues in the transmembrane and intracellular domain are highlighted with pink color. *$p<0.01$, **$p<0.01$, ***$p<0.001$.

Figure S3

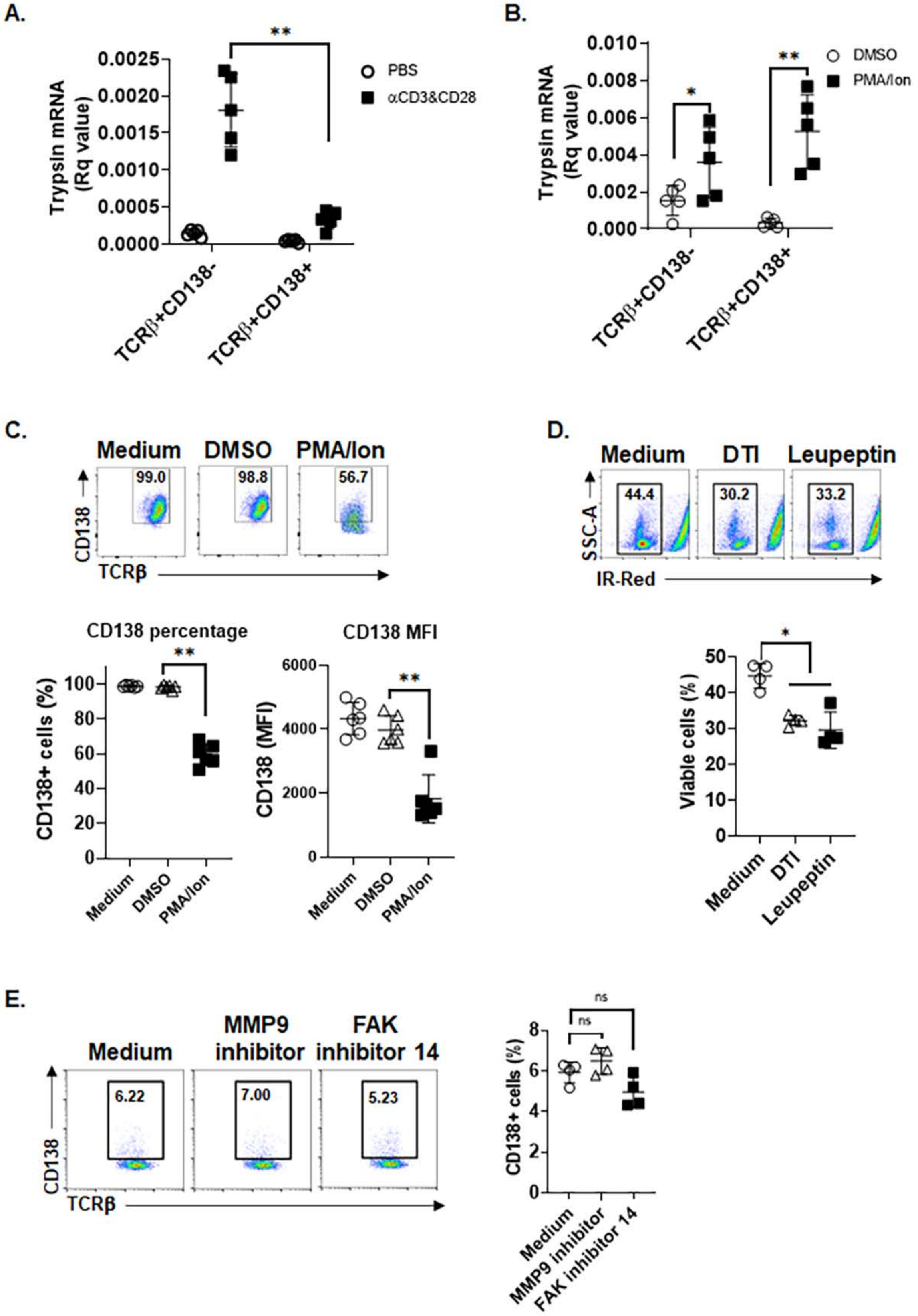
A.
**
Trypsin mRNA (Rq value)
0.0025
0.0020
0.0015
0.0010
0.0005
0.0000
PBS
αCD3&CD28
TCRβ+CD138-
TCRβ+CD138+
B.
Trypsin mRNA (Rq value)
0.010
0.008
0.006
0.004
0.002
0.000
*
**
DMSO
PMA/Ion
TCRβ+CD138-
TCRβ+CD138+
C.
Medium
DMSO
PMA/Ion
99.0
98.8
56.7
CD138
TCRβ
CD138 percentage
CD138 MFI
CD138+ cells (%)
CD138 (MFI)
Medium
DMSO
PMA/Ion
D.
Medium
DTI
Leupeptin
44.4
30.2
33.2
SSC-A
IR-Red
Viable cells (%)
Medium
DTI
Leupeptin
E.
Medium
MMP9 inhibitor
FAK inhibitor 14
6.22
7.00
5.23
CD138
TCRβ
ns
CD138+ cells (%)
Medium
MMP9 inhibitor
FAK inhibitor 14

Figure S3. Trypsin expression is increased in activated T cells.

A - C, purified TCRβ+CD138+ and TCRβ+CD138- cells from 10 to 12 weeks old MRL/Lpr mice were treated with αCD3/CD28 antibodies (***A***) or PMA and Ionomycin (PMA/Ion) **(*B*)** for 48 hours, and then trypsin mRNA levels were measured by Q-PCR or surface CD138 expression **(*C*)** was measured by FACS. Mean ± SD of five to six different samples are plotted. ***D***, purified TCRβ+CD138- cells from 10 to 12 weeks old MRL/Lpr mice were cultured in serum free medium or treated with trypsin inhibitors DTI and Leupeptin for 24 hours, after which cell-survival was measured by FACS. Mean ± SD of four different samples are plotted. ***E***, Purified TCRβ+CD138- cells from 10 to 12 weeks old MRL/Lpr mice were cultured in serum free medium or treated with MMP9 specific inhibitor APMA and FAK inhibitor 14 for 24 hours, after which CD138+ cells were measured by FACS. Mean ± SD of four different samples are plotted. ns, not significant. $^*p<0.05$, $^{**}p<0.01$.

Figure S4

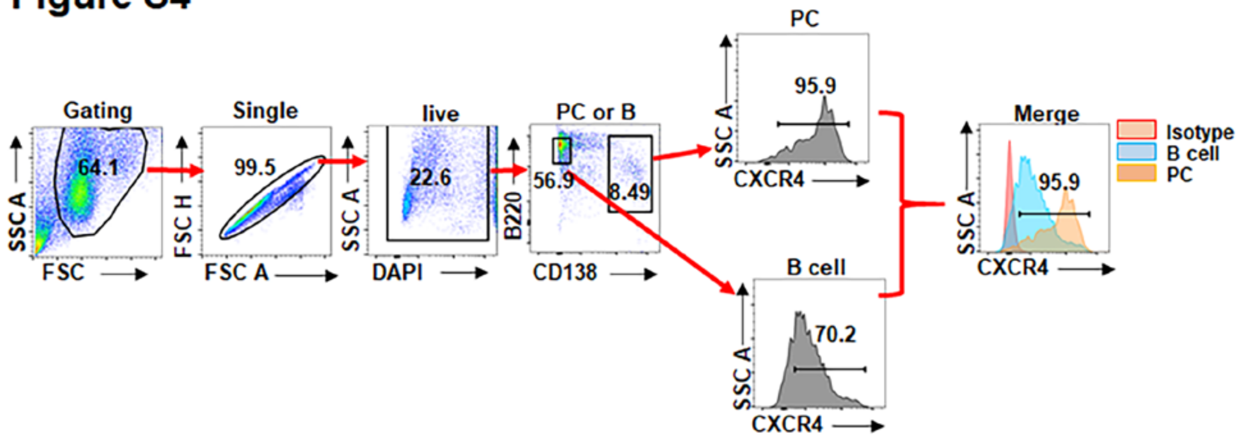

Gating
64.1
SSC A
FSC
Single
99.5
FSC H
FSC A
live
22.6
SSC A
DAPI
PC or B
56.9
8.49
B220
CD138
PC
95.9
SSC A
CXCR4
B cell
70.2
SSC A
CXCR4
Merge
95.9
SSC A
CXCR4
Isotype
B cell
PC

Figure S4. Gating strategy for measuring CXCR4 on plasma cells and B cells.
The singlet and live cells were gated before staining with B220 and CD138 antibodies in FACS. Expression of CXCR4 on plasma cells and B cells were for further analyzed by FACS.

Figure S5

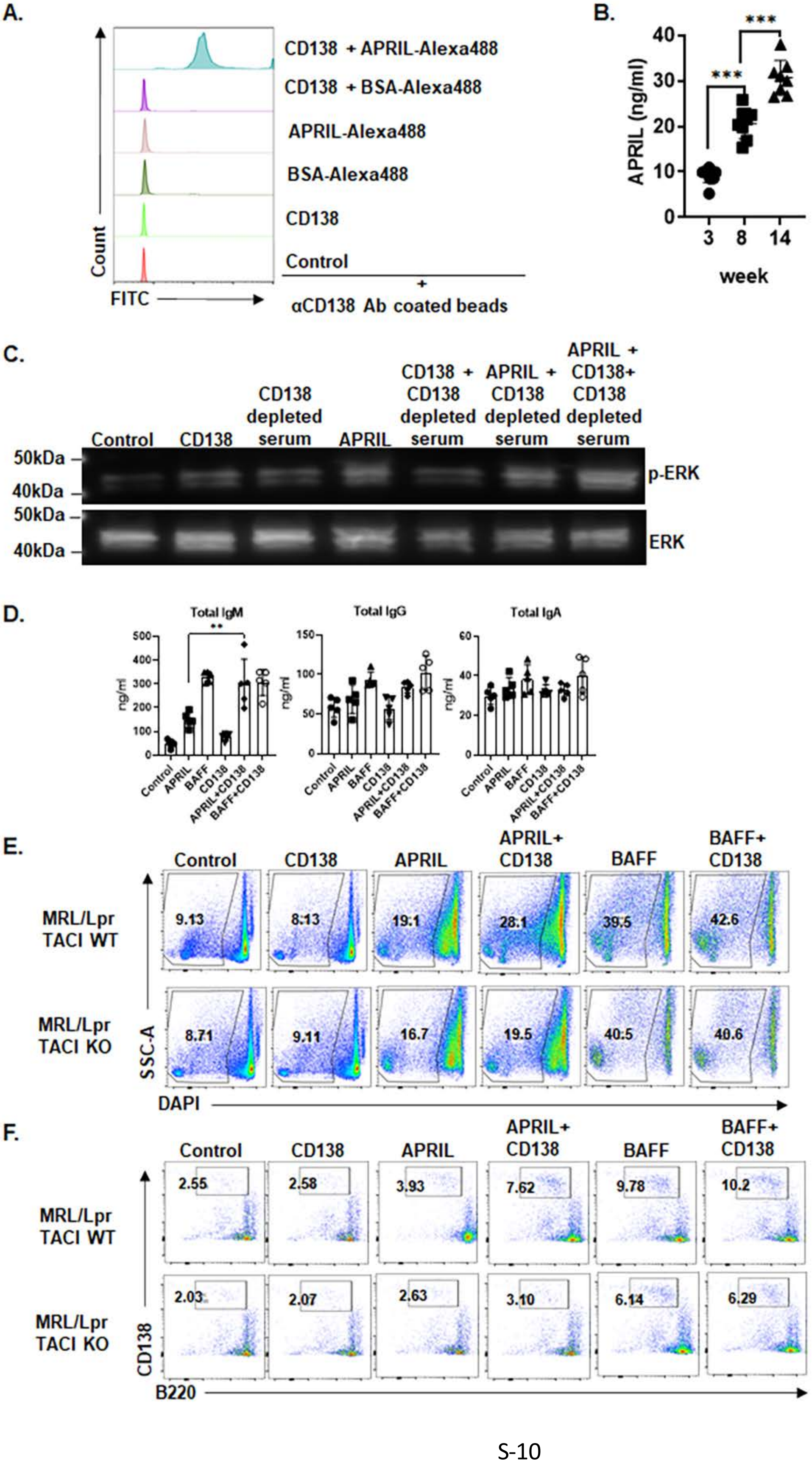
A.
CD138 + APRIL-Alexa488
CD138 + BSA-Alexa488
APRIL-Alexa488
BSA-Alexa488
CD138
Control
+
αCD138 Ab coated beads
Count
FITC
B.
APRIL (ng/ml)
BAFF (ng/ml)
week
C.
Control
CD138
CD138 depleted serum
APRIL
CD138 + CD138 depleted serum
APRIL + CD138 depleted serum
APRIL + CD138+ CD138 depleted serum
50kDa
40kDa
p-ERK
ERK
D.
Total IgM
Total IgG
Total IgA
ng/ml
Control
APRIL
BAFF
CD138
APRIL+CD138
BAFF+CD138
E.
Control
CD138
APRIL
APRIL+ CD138
BAFF
BAFF+ CD138
MRL/Lpr TACI WT
MRL/Lpr TACI KO
9.13
8.13
19.1
28.1
39.5
42.6
8.71
9.11
16.7
19.5
40.5
40.6
SSC-A
DAPI
F.
Control
CD138
APRIL
APRIL+ CD138
BAFF
BAFF+ CD138
MRL/Lpr TACI WT
MRL/Lpr TACI KO
2.55
2.58
3.93
7.62
9.78
10.2
2.03
2.07
2.63
3.10
6.14
6.29
CD138
B220

Figure S5. CD138 interacts with APRIL and enhances APRIL-induced ERK phosphorylation and B cell survival through TACI.

A, protein A and G Sepharose beads were coated with anti-CD138 antibody first, and then incubated with soluble CD138, BSA-Alexa 488 or APRIL-Alexa488 alone or in combination. After incubation at room temperature for 1 hour while shaking, beads were washed and subjected to FACS analysis. Histogram images from one representative experiment out of three independent experiments are shown. ***B***, sera were collected from MRL/Lpr mice at different ages and the serum APRIL and BAFF levels were measured by ELISA. Mean ± SD of 8 to 11 different samples are plotted. ***C***, MRL/Lpr B cells were left in 2% FBS or treated with CD138, CD138 depleted serum, APRIL alone, CD138 plus CD138 depleted serum, CD138 depleted serum plus APRIL or CD138 plus CD138 depleted serum plus APRIL for overnight and ERK phosphorylation was detected with Western Blot analysis. ***D***, MRL/Lpr B cells were treated with APRIL, BAFF, CD138, APRIL plus CD138 or BAFF plus CD138 for 5 days, and culture supernatant total IgM, IgG and IgA antibody levels were quantified in ELISA. Mean ± SD of four independent experiments are plotted. ***E***, B cells isolated from MRL/Lpr TACI WT and MRL/Lpr TACI KO mice were cultured in 10% FBS and treated with CD138, APRIL, APRIL plus CD138, BAFF or BAFF plus CD138 for 5 days, and B cell survival was measured with FACS analysis. Representative images from six independent experiments are shown. ***F***, B cells isolated from MRL/Lpr TACI WT and MRL/Lpr TACI KO mice were cultured in 10% FBS and treated with CD138, APRIL, APRIL plus CD138, BAFF or BAFF plus CD138 for 5 days, and plasma cell formation was measured with FACS analysis. Representative images from six independent experiments are shown. *p<0.05, **p<0.01, ***p<0.001.
